# The Association of Placental Grading with Perinatal Outcomes: A Systematic Review and Meta-Analysis

**DOI:** 10.3390/diagnostics15101264

**Published:** 2025-05-15

**Authors:** Antonios Siargkas, Christina Pachi, Meletios P. Nigdelis, Sofoklis Stavros, Ekaterini Domali, Apostolos Mamopoulos, Ioannis Tsakiridis, Themistoklis Dagklis

**Affiliations:** 1 Third Department of Obstetrics and Gynecology, School of Medicine, Faculty of Health Sciences, Aristotle University of Thessaloniki, Agiou Dimitriou, 54124 Thessaloniki, Greece; antonis.siargkas@gmail.com (A.S.);; 2 Department of Gynecology, Obstetrics and Reproductive Medicine, Saarland University Medical Center (UKS), 66421 Homburg, Germany; 3 Third Department of Obstetrics and Gynecology, University Hospital “ATTIKON”, Medical School, National and Kapodistrian University of Athens, 12462 Athens, Greece; 4 First Department of Obstetrics and Gynecology, Alexandra Hospital Athens, 11528 Athens, Greece

**Keywords:** placental calcification, Grannum grading, small-for-gestational-age, fetal growth restriction, preeclampsia, stillbirth

## Abstract

**Objective**: Premature placental calcification (PPC) has been implicated in adverse perinatal outcomes, yet its clinical significance remains controversial. This meta-analysis aimed to quantitatively synthesize current data on the association between PPC, defined as grade 3 placental calcification before 36^+6^ weeks of gestation and adverse perinatal outcomes. Data Sources: A systematic search was conducted in MEDLINE, Scopus and The Cochrane Library from inception until 11 March 2025, to identify eligible studies. Study Eligibility Criteria: Observational studies including singleton pregnancies with PPC diagnosed via ultrasonography between 28^+0^ and 36^+6^ weeks of gestation and comparing them with pregnancies with Grannum grade 0, 1, or 2 placentas were considered eligible. **Methods**: Study quality was assessed using the Newcastle−Ottawa Scale, and the risk of bias was evaluated with the Quality In Prognosis Studies tool. The primary outcomes were small-for-gestational-age (SGA) neonates and preeclampsia. Heterogeneity was assessed using Cochran’s Q test and the I^2^ statistic. Meta-analyses were conducted using a random-effects model, with outcomes reported as relative risk (RR) or mean difference (MD) with 95% confidence intervals (CIs). **Results**: In total, nine cohort studies were included. PPC was associated with an increased risk of SGA (RR, 1.99; 95% CI, 1.46−2.70), preeclampsia (RR, 5.27; 95% CI, 2.24−12.40), fetal growth restriction (RR, 2.31; 95% CI, 1.30−4.09), preterm delivery (RR, 2.11; 95% CI, 1.00−4.45), suspected fetal hypoxia (RR, 1.71; 95% CI, 1.13–2.56), low 5 min Apgar score (RR, 2.28; 95% CI, 1.50−3.44) and neonatal intensive care unit admission (RR, 1.80; 95% CI, 1.02−3.18). No significant associations were found with fetal or neonatal death (RR, 2.75; 95% CI, 0.87−8.71), cesarean delivery (RR, 1.26; 95% CI, 0.90−1.78), gestational diabetes mellitus (RR, 1.17; 95% CI, 0.81−1.70), neonatal resuscitation (RR, 1.04; 95% CI, 0.92−1.16), birthweight (MD, −187.46 g; 95% CI, −413.14 to +38.21), or gestational age at birth (MD, −0.62 weeks; 95% CI, −1.36 to +0.11). A sensitivity analysis excluding high-risk-of-bias studies yielded consistent results. **Conclusions**: PPC is associated with several adverse perinatal outcomes, including SGA and preeclampsia. While the clinical significance of placental grading has remained limited in recent years, this study has shown that PPC may serve as an early indicator of placental insufficiency, warranting enhanced fetal surveillance and risk assessment in affected pregnancies. Further research is needed to refine its prognostic utility and integration into obstetric practice.

## 1. Introduction

As pregnancy progresses, the placenta undergoes structural changes visible on ultrasound, primarily due to increasing calcification; this phenomenon was first associated with fetal maturity by Winsberg [1]. Following that observation, a grading system had been developed to categorize placental maturation; grade 3 placental calcification was initially linked to fetal pulmonary maturity at birth [2]. As research on placental grading progressed, premature placental calcification (PPC), defined as grade 3 calcification occurring before 36 weeks of gestation, had been implicated in various pregnancy complications, including preeclampsia [3,4], fetal growth restriction (FGR) [3,4,5,6], stillbirth [7] and low Apgar score (<7 at 5 min) [4]. However, the clinical significance of PPC remains controversial, as several subsequent studies have failed to replicate previously reported associations [4,6,8,9].

The most recent meta-analysis on this subject, published in 2018, did not specifically focus on PPC, lacked gestational age restrictions for placental grading and applied broad inclusion criteria, thereby limiting the generalizability of its findings [10]. This analysis suggested that pregnancies with a grade 3 placenta may be at increased risk of perinatal death, meconium-stained amniotic fluid and low birth weight, but did not investigate other key adverse outcomes such as FGR and preeclampsia [10]. Furthermore, research interest in placental grading has declined in recent years, possibly due to inconsistencies in its evaluation across sonographers and the ambiguity of its clinical significance. It has been assumed that uterine and umbilical artery pulsatility index (UA, UtA PI) assessment, when available, adequately describe the feto-placental interaction.

Nevertheless, a recent retrospective cohort study, which accounted for multiple confounders, including UtA PI percentile, identified a significant association between PPC and both small-for-gestational-age (SGA) neonates and FGR [11]. Additionally, while this study also suggested a positive trend, it did not find a statistically significant association with stillbirth or preeclampsia in the multivariable models [11]. These findings suggest that PPC may have clinical value as an independent prognostic factor for certain adverse outcomes.

The mechanisms by which PPC may negatively impact perinatal outcomes could involve narrowing placental blood vessels, potentially through atherosclerosis, basement membrane mineralization, or nanobacteria [12,13]. Furthermore, cellular processes linked to aging, like gene expression changes and hormonal imbalances, specifically, dysregulation of the hypothalamic–pituitary–adrenal axis and elevated cortisol, might also play a role in placental aging and related complications [14,15].

Building on these findings, this meta-analysis aimed to comprehensively synthesize the literature to determine the precise effect estimates of PPC on perinatal outcomes. By examining discrepancies across studies and clarifying the implications of Grannum grading, we attempted to elucidate the clinical role of PPC and offer insights into current obstetric practice.

## 2. Methods

The study’s design and reporting adhered to the Preferred Reporting Items for Systematic reviews and meta-analyses (PRISMA) [16]. The study’s protocol was officially documented in the PROSPERO registry under the identifier CRD42024626749. Since this investigation relied solely on data already available in published form, neither ethical board approval nor individual patient consent was deemed necessary.

### 2.1. Search Strategy

To address the primary research question, “Is premature placental calcification associated with adverse perinatal outcomes?” we developed a comprehensive search strategy based on the PICO framework. We systematically searched three major databases: MEDLINE, Scopus, and The Cochrane Library, from their inception up to 11 March 2025. The search incorporated keywords related to “placental senescence”, “placental calcification”, “Grannum grading”, “placental grading” and associated terms. The detailed search strategy is provided in the Supplementary Materials.

All identified records were imported into Rayyan (Rayyan Systems Inc, Cambridge, MA), an online tool for managing and screening references. Following the removal of duplicate entries, two independent reviewers (A.S. and C.P.—both obstetricians) screened the titles and abstracts to exclude studies that did not meet the inclusion criteria. The full texts of the remaining articles were then meticulously reviewed to confirm eligibility. Discrepancies between reviewers were resolved through discussion, and if necessary, a third reviewer (I.T.—biostatistician) was consulted to reach a consensus.

Additionally, we performed a manual review of the reference lists of all pertinent studies and conducted supplementary online searches to identify any further eligible studies. Only studies published in English were considered, and there were no restrictions based on publication date.

### 2.2. Selection Criteria

Eligible studies encompassed prospective and retrospective cohort studies, case-control studies, and controlled trials that examined singleton pregnancies with live fetuses undergoing placental grading between 28^+0^ and 36^+6^ weeks of gestation. The exposure of interest was defined as the presence of a Grannum grade 3 placenta, detected via ultrasonography within this gestational window. The control group comprised pregnancies with placentas classified as Grannum grades 0, 1, or 2, assessed between the 28^+0^ and 36^+6^ weeks of gestation.

Studies were excluded if they did not provide raw data on maternal or perinatal outcomes, involved multiple gestations, reported on pregnancies with known genetic anomalies or major fetal defects (identified either prenatally or postnatally), or were limited to abstracts, conference proceedings, or case reports. Additionally, studies where the primary exposure or outcomes were amalgamated in a manner that precluded separate analysis were excluded.

### 2.3. Data Extraction

A standardized datasheet was developed prior to the screening process to ensure consistency in data collection. Two reviewers (A.S. and C.P.) independently extracted the following information from each eligible study:

- Study Characteristics: Author(s), year of publication, journal name, country of study, study design (prospective/retrospective cohort, case-control, or controlled trial) and study duration.
- Population Details: Inclusion and exclusion criteria, definition and timing of PPC and description of the control group.
- Outcomes Reported: Raw data on adverse perinatal outcomes, where available, adjusted measures like adjusted odds ratios (aORs) or adjusted risk ratios (aRRs) were also extracted.

In instances where multiple publications reported on the same cohort, data from the most comprehensive or recent study were utilized. Should essential data be missing, corresponding authors were contacted for clarification. Any disagreements during data extraction were resolved through discussion or by involving a third reviewer (I.T.).

### 2.4. Quality and Risk of Bias Assessment

The methodological quality of the included studies was independently evaluated by two reviewers (A.S. and C.P.) using the Newcastle–Ottawa Scale (NOS) [17]. This scale assesses observational studies based on three domains: selection of study groups, comparability of groups and ascertainment of outcomes. Each study was awarded stars corresponding to its quality, with a maximum possible score of nine stars indicating the highest quality.

To further evaluate the risk of bias in prognostic factor studies, the Quality In Prognosis Studies (QUIPS) tool was employed and its results are displayed next to the forest plots [18]. QUIPS examines six domains: (a) Study Participation, (b) Study Attrition, (c) Prognostic Factor Measurement, (d) Outcome Measurement, (e) Study Confounding and (f) Statistical Analysis and Reporting. Each domain was rated for risk of bias as low, moderate, or high. Discrepancies between reviewers were addressed through discussion or by consulting a third reviewer (I.T.).

### 2.5. Statistical Analysis

All statistical analyses were conducted using Review Manager (RevMan, version 5.4.1; Cochrane, London, UK) for primary calculations and RStudio version 4.4.2 (RStudio, Boston, MA, USA) for assessing publication bias. For dichotomous outcomes, risk ratios (RRs) with 95% confidence intervals (CIs) were calculated using the Mantel–Haenszel method. Continuous outcomes were analyzed by estimating the mean difference (MD) with corresponding 95% CIs using the inverse–variance method.

Given the anticipated heterogeneity among observational studies, the DerSimonian and Laird random-effects model was exclusively utilized. Heterogeneity was assessed using Cochran’s Q test (with a significance threshold of *p* < 0.1) and the I^2^ statistic, which quantifies the percentage of total variation across studies attributable to heterogeneity rather than chance [19]. Publication bias was evaluated visually through funnel plots and quantitatively using Egger’s test in RStudio [20].

### 2.6. Sensitivity Analyses

A sensitivity analysis was performed by excluding studies identified as having a serious risk of bias according to the QUIPS tool. Additionally, we intended to conduct sensitivity analyses using only adjusted effect measures (aORs or aRRs) if at least three studies per outcome provided these estimates. However, this was not feasible due to insufficient data.

### 2.7. Outcomes

In alignment with our predefined protocol, the primary outcomes of interest were SGA neonates, defined as birthweight below the 10th percentile and preeclampsia [21]. Secondary outcomes encompassed a range of adverse perinatal outcomes, including FGR, stillbirth, preterm delivery (<37 weeks), cesarean delivery, birthweight, admission to the neonatal intensive care unit (NICU), 5 min Apgar scores below 7 and other relevant adverse pregnancy outcomes with sufficient data availability (more than 2 studies).

## 3. Results

### 3.1. Search Results and Study Selection

**Figure 1:**
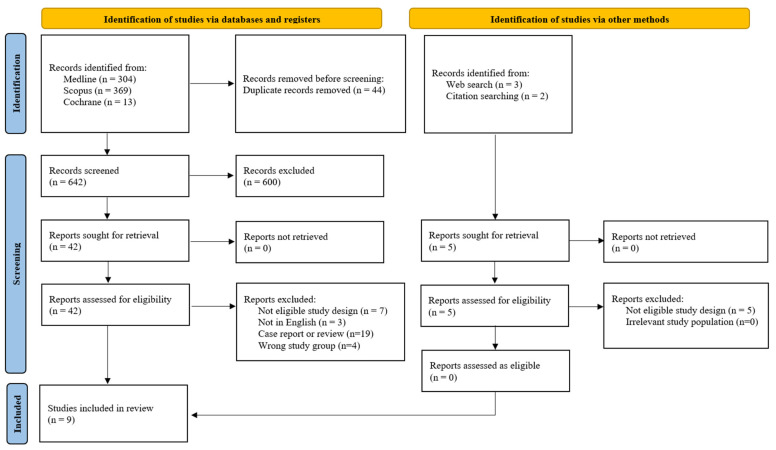
Study selection flow diagram.

The initial search yielded a total of 686 articles. Following the removal of 44 duplicate entries, the remaining 642 records underwent screening of their titles and abstracts, leading to the exclusion of 600 irrelevant studies. Subsequently, the full text of the remaining 42 studies was thoroughly examined to determine their suitability for inclusion. Furthermore, a review of the reference lists of all pertinent studies, supplemented by an additional online search, identified another five potentially relevant articles. Three studies that might appear to meet the inclusion criteria were excluded from the analysis due to the following reasons: one study reported a composite score for adverse outcomes rather than raw data for every outcome [22], another utilized ex vivo ultrasound on the placenta post-partum [23] and a third assessed placental grading between 37 and 41 weeks of gestation, thus falling outside the defined criteria for PPC [24]. Ultimately, nine cohort studies satisfied the inclusion criteria and were incorporated into this meta-analysis [3,4,5,7,8,9,11,25,26], as visually outlined in Figure 1. Key characteristics of the studies that met the inclusion criteria are detailed in Table 1.

### 3.2. Quality Assessment of the Included Studies

All included studies were cohort studies, comprising eight prospective and one retrospective cohort. Quality assessment was conducted using the Newcastle–Ottawa Scale (Table 2). Two prospective cohort studies and the single retrospective cohort study received the highest possible rating of nine stars. Among the remaining prospective studies, five scored seven stars, while one received six stars due to limitations primarily in the comparability domain. No other significant sources of bias were detected.

## 4. Synthesis of Results

### 4.1. Analysis of Dichotomous Outcomes

#### 4.1.1. Small-for-Gestational-Age Neonates

**Figure 2:**
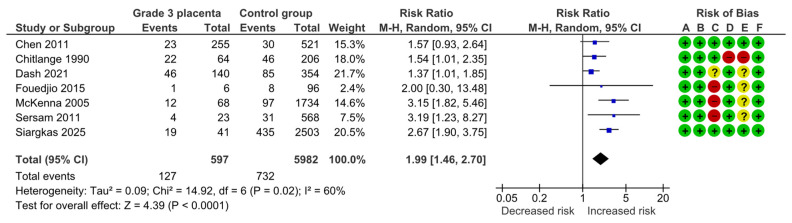
Forest plot investigating the risk of small-for-gestational-age neonates in singleton pregnancies with preterm placental calcification [3,4,5,8,9,11,25]. Abbreviations: CI, confidence interval; M–H, Mantel–Haenszel method.

Seven studies assessed SGA, including 597 pregnancies with grade 3 placentas and 5982 controls. Overall, 127 (21.3%) cases of SGA were detected in the grade 3 group compared with 732 (12.2%) in controls, yielding a significantly increased risk for SGA (RR, 1.99; 95% CI, 1.46–2.70; I^2^ = 60%) (Figure 2).

**Figure 3:**
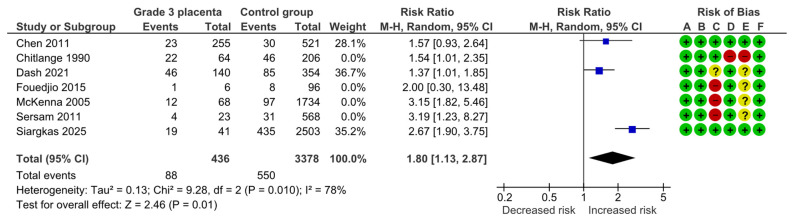
Forest plot investigating the risk of small-for-gestational-age neonates in singleton pregnancies with preterm placental calcification after excluding the high risk of bias studies [3,4,5,8,9,11,25]. Abbreviations: CI, confidence interval; M–H, Mantel–Haenszel method.

After excluding those studies deemed as high risk of bias, three studies remained with 436 study and 3378 control population. The sensitivity analysis also showed a significant association (RR, 1.80; 95% CI, 1.13–2.87; I^2^ = 78%) (Figure 3).

#### 4.1.2. Fetal Growth Restriction

**Figure 4:**
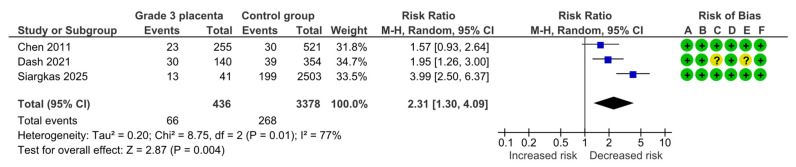
Forest plot investigating the risk of fetal growth restriction in singleton pregnancies with preterm placental calcification [4,11,25]. Abbreviations: CI, confidence interval; M–H, Mantel–Haenszel method.

Three studies assessed FGR, including 436 pregnancies with grade 3 placentas and 3378 controls. Overall, 66 (15.1%) cases of FGR were detected in the grade 3 group compared with 268 (7.9%) in controls, yielding a significantly increased risk for FGR (RR, 2.31; 95% CI, 1.30–4.09; I^2^ = 77%) (Figure 4).

#### 4.1.3. Preeclampsia

**Figure 5:**
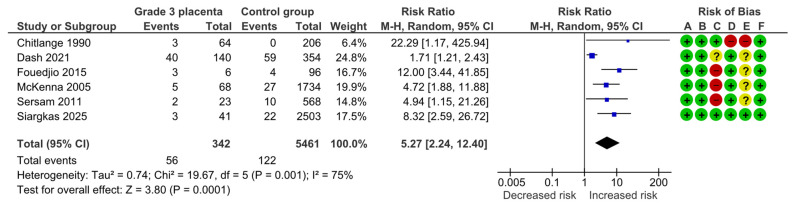
Forest plot investigating the risk of preeclampsia in singleton pregnancies with preterm placental calcification [3,4,5,8,9,11]. Abbreviations: CI, confidence interval; M–H, Mantel–Haenszel method.

Six studies assessed preeclampsia, including 342 pregnancies with grade 3 placentas and 5461 controls. Overall, 56 (16.4%) cases of preeclampsia were detected in the grade 3 group compared with 122 (2.2%) in controls, yielding a significantly increased risk for preeclampsia (RR, 5.27; 95% CI, 2.24–12.40; I^2^ = 75%) (Figure 5).

#### 4.1.4. Fetal or Neonatal Death

**Figure 6:**
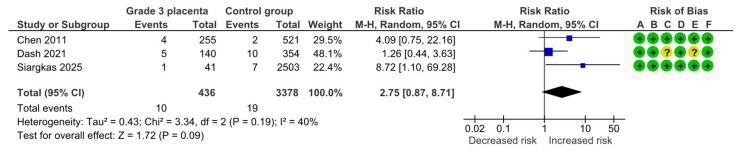
Forest plot investigating the risk of fetal or neonatal death in singleton pregnancies with preterm placental calcification [4,11,25]. Abbreviations: CI, confidence interval; M–H, Mantel–Haenszel method.

Three studies assessed fetal or neonatal deaths, including 436 pregnancies with grade 3 placentas and 3378 controls. Overall, 10 (2.3%) deaths were detected in the grade 3 group compared with 19 (0.6%) in controls, yielding no statistically significant difference for fetal or neonatal deaths (RR, 2.75; 95% CI, 0.87–8.71; I^2^ = 40%) (Figure 6).

#### 4.1.5. Preterm Delivery

**Figure 7:**
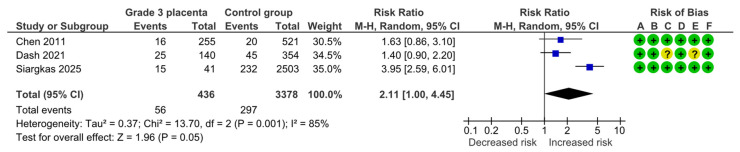
Forest plot investigating the risk of preterm delivery in singleton pregnancies with preterm placental calcification [4,11,25]. Abbreviations: CI, confidence interval; M–H, Mantel–Haenszel method.

Three studies assessed preterm delivery, including 436 pregnancies with grade 3 placentas and 3378 controls. Overall, 56 (12.8%) cases of preterm delivery were detected in the grade 3 group compared with 297 (8.8%) in controls, yielding a significantly increased risk for preterm delivery (RR, 2.11; 95% CI, 1.00–4.45; I^2^ = 85%) (Figure 7).

#### 4.1.6. Cesarean Section

**Figure 8:**
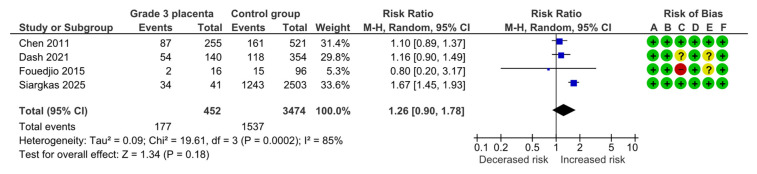
Forest plot investigating the risk of cesarean section in singleton pregnancies with preterm placental calcification [4,9,11,25]. Abbreviations: CI, confidence interval; M–H, Mantel–Haenszel method.

Four studies assessed cesarean section, including 452 pregnancies with grade 3 placentas and 3474 controls. Overall, 177 (39.2%) cases of cesarean delivery were detected in the grade 3 group compared with 1537 (44.2%) in controls, yielding no statistically significant difference for cesarean section (RR, 1.26; 95% CI, 0.90–1.78; I^2^ = 85%) (Figure 8).

**Figure 9:**
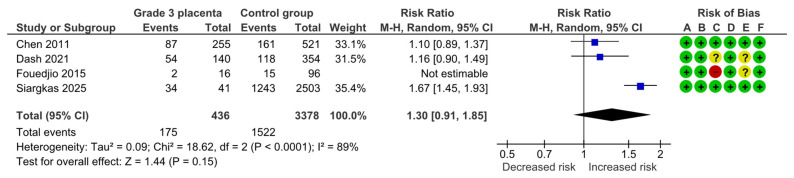
Forest plot investigating the risk of cesarean section in singleton pregnancies with preterm placental calcification after excluding high risk of bias studies [4,9,11,25]. Abbreviations: CI, confidence interval; M–H, Mantel–Haenszel method.

After excluding those studies deemed as high risk of bias, three studies remained with 436 vs. 3378 pregnancies. The sensitivity analysis also yielded a non-significant association (RR, 1.30; 95% CI, 0.91–1.85; I^2^ = 89%) (Figure 9).

#### 4.1.7. Low 5-Minute Apgar Score

**Figure 10:**
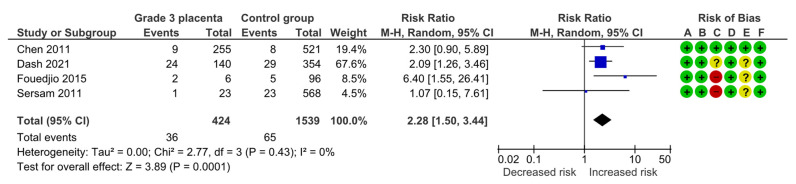
Forest plot investigating the risk of low 5 min Apgar score in singleton pregnancies with preterm placental calcification [4,8,9,25]. Abbreviations: CI, confidence interval; M–H, Mantel–Haenszel method.

Four studies assessed low 5 min Apgar scores (<7), including 424 pregnancies with grade 3 placentas and 1539 controls. Overall, 36 (8.5%) cases of low 5 min Apgar were detected in the grade 3 group compared with 65 (4.2%) in controls, yielding a significantly increased risk for a low 5 min Apgar score (RR, 2.28; 95% CI, 1.50–3.44; I^2^ = 0%) (Figure 10).

#### 4.1.8. Neonatal Intensive Care Unit Admission

**Figure 11:**
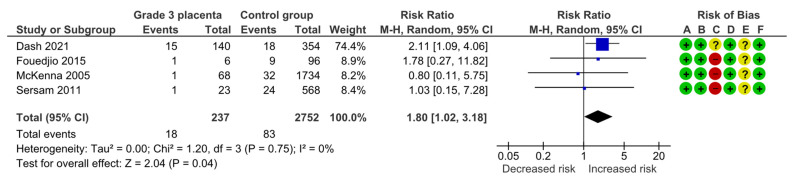
Forest plot investigating the risk of neonatal intensive care unit admission in singleton pregnancies with preterm placental calcification [3,4,8,9]. Abbreviations: CI, confidence interval; M–H, Mantel–Haenszel method.

Four studies assessed NICU admission including 237 pregnancies with grade 3 placentas and 2752 controls. Overall, 18 (7.6%) NICU admissions were detected in the grade 3 group compared with 83 (3.0%) in controls, yielding a significantly increased risk for NICU admission (RR, 1.80; 95% CI, 1.02–3.18; I^2^ = 0%) (Figure 11).

#### 4.1.9. Neonatal Resuscitation

**Figure 12:**
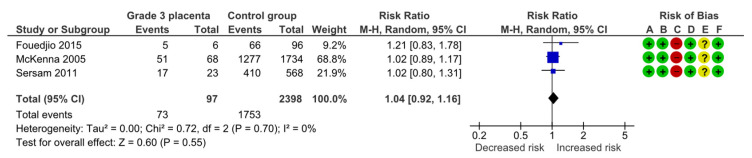
Forest plot investigating the risk of neonatal resuscitation in singleton pregnancies with preterm placental calcification [3,8,9]. Abbreviations: CI, confidence interval; M–H, Mantel–Haenszel method.

Three studies assessed neonatal resuscitation, including 97 pregnancies with grade 3 placentas and 2398 controls. Overall, 73 (75.3%) cases requiring neonatal resuscitation were detected in the grade 3 group compared with 1753 (73.1%) in controls, yielding no statistically significant difference for neonatal resuscitation (RR, 1.04; 95% CI, 0.92–1.16; I^2^ = 0%) (Figure 12).

#### 4.1.10. Gestational Diabetes Mellitus

**Figure 13:**
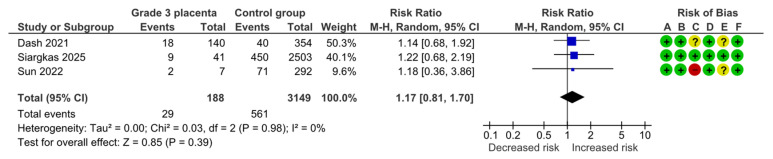
Forest plot investigating the risk of gestational diabetes mellitus in singleton pregnancies with preterm placental calcification [4,11,26]. Abbreviations: CI, confidence interval; M–H, Mantel–Haenszel method.

Three studies assessed gestational diabetes mellitus (GDM), including 188 pregnancies with grade 3 placentas and 3149 controls. Overall, 29 (15.4%) cases of GDM were detected in the grade 3 group compared with 561 (17.8%) in controls, yielding no statistically significant difference for GDM (RR, 1.17; 95% CI, 0.81–1.70; I^2^ = 0%) (Figure 13).

#### 4.1.11. Suspected Fetal Hypoxia

**Figure 14:**
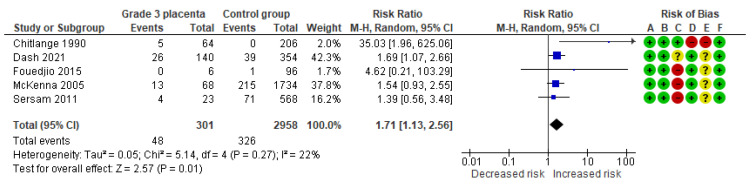
Forest plot investigating the risk of suspected fetal hypoxia in singleton pregnancies with preterm placental calcification [3,4,5,8,9]. Abbreviations: CI, confidence interval; M–H, Mantel–Haenszel method.

Five studies assessed suspected fetal hypoxia, including 301 pregnancies with grade 3 placentas and 2958 controls. Overall, 48 (15.9%) cases of suspected fetal hypoxia were detected in the grade 3 group compared with 326 (11%) in controls, yielding a significantly increased risk for suspected fetal hypoxia (RR, 1.71; 95% CI, 1.13–2.56; I^2^ = 22%) (Figure 14).

### 4.2. Analysis of Continuous Outcomes

#### 4.2.1. Birthweight

**Figure 15:**
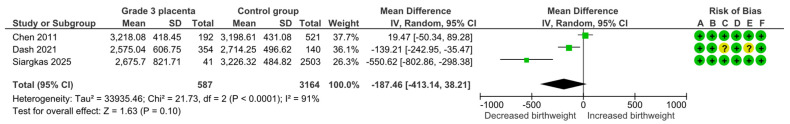
Forest plot investigating the association of birthweight with preterm placental calcification in singleton pregnancies [4,11,25]. Abbreviations: CI, confidence interval; M–H, Mantel–Haenszel method.

Three studies assessed birthweight, including 587 pregnancies with grade 3 placentas and 3164 controls. Overall, the pooled mean difference was −187.46 g (95% CI, −413.14 to +38.21g; I^2^ = 91%), indicating no statistically significant difference in birthweight (Figure 15).

#### 4.2.2. Gestational Age at Birth

**Figure 16:**
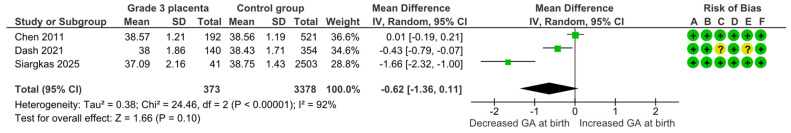
Forest plot investigating the association of gestational age at birth with preterm placental calcification in singleton pregnancies [4,11,25]. Abbreviations: CI, confidence interval; M–H, Mantel–Haenszel method.

Three studies assessed gestational age at birth, including 373 pregnancies with grade 3 placentas and 3378 controls. Overall, the pooled mean difference was −0.62 weeks (95% CI, −1.36 to +0.11 weeks; I^2^ = 92%) indicating no statistically significant difference in gestational age at birth (Figure 16).

The cumulative results of our meta-analyses are summarized in Table 3.

### 4.3. Publication Bias

**Figure 17:**
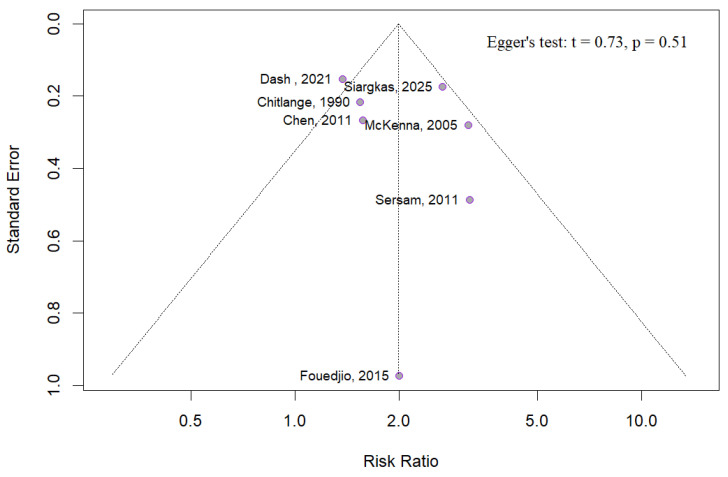
Funnel plot and Egger’s test for the small-for-gestational-age meta-analysis [3,4,5,8,9,11,25].

An evaluation for publication bias regarding the primary outcome (SGA), was conducted. Visual inspection of the funnel plot, along with the result of Egger’s test (*p* = 0.51), provided no evidence to suggest the presence of publication bias (Figure 17). Nevertheless, it is important to consider that the meta-analysis for SGA included only seven studies, potentially limiting the ability of both the funnel plot and the Egger test to definitively identify publication bias due to insufficient statistical power.

## 5. Discussion

### 5.1. Primary Findings

This meta-analysis showed that pregnancies complicated by PPC are at increased risk for SGA, FGR, preeclampsia, suspected fetal hypoxia, preterm delivery, low 5-min Apgar score and NICU admission. No significant associations were detected with fetal or neonatal death, gestational diabetes mellitus, cesarean delivery, neonatal resuscitation, birthweight, or gestational age at birth.

### 5.2. Interpretation of the Findings

This meta-analysis demonstrated a two-fold increase in the risk of FGR and SGA in pregnancies affected by PPC. While there was significant heterogeneity among the included studies, all of them, including those that did not reach statistical significance, consistently indicated a positive association with both adverse outcomes. Additionally, the findings were reinforced by our risk-of-bias sensitivity analysis, which further confirmed the robustness of this association after the exclusion of the high-risk of bias studies. Notably, the most recent study on this topic reported statistically significant associations between PPC and both SGA and FGR, after adjusting for multiple confounders, including the UtA PI [11]. This finding is particularly important as it suggests that PPC may serve as an independent prognostic factor for these adverse outcomes, underscoring the need for further research in this area. A plausible explanation for these observed links is that PPC may impair placental function by restricting blood flow and nutrient delivery to the fetus. Calcium and fibrin deposits may obstruct placental vasculature, reducing circulation. This is evident in fetal Bartter syndrome, where mineral accumulation in the basement membrane coincides with calcification and atherosclerotic changes in placental vessels [13,27]. Calcifications in the basal plate are also linked to maternal floor infarction [28], a condition associated with FGR and mid-trimester pregnancy loss [29]. Furthermore, calcifications have been observed obstructing chorionic and umbilical vessels, strengthening the connection between PPC and FGR [30,31]. These findings suggest PPC may not be a benign pregnancy feature, but rather a pathological factor associated with adverse outcomes. At the molecular level, placental aging may contribute to SGA and FGR through telomere shortening, mitochondrial dysfunction and oxidative stress, leading to impaired nutrient transport and increased apoptosis via elevated p53 levels [32]. Angiogenic imbalance, indicated by decreased Placental Growth Factor (PlGF) and an elevated sFlt-1/PlGF ratio, strongly correlates with placental insufficiency and SGA risk [33]. Low Pregnancy-Associated Plasma Protein-A levels further signal impaired placental function [33], while oxidative stress markers like Hypoxia-Inducible Factor 1-alpha are linked to telomere erosion and accelerated cellular aging [34].

Singleton pregnancies affected by PPC were associated with a five-fold increased risk of preeclampsia. However, this contrasts with the findings of the most recently published retrospective study, which showed that, after adjusting for significant confounders, including the uterine artery pulsatility index, in a multivariable logistic regression, the association between PPC and preeclampsia was no longer significant [11]. These results suggest that while PPC is linked to preeclampsia, it may not serve as a major individual prognostic factor, with the uterine artery pulsatility index also being an important predictor.

Regarding fetal and neonatal death, statistical significance was not reached; two relevant studies explored this relationship after adjusting for confounders. The first study, which included 15,122 singleton pregnancies, adjusted for maternal age, body mass index and parity, identifying a significant association with an aOR of 7.62 (95% CI 5.0–11.6). In contrast, the second study, involving 3088 pregnancies, adjusted for additional factors such as assisted reproductive technology, smoking and uterine artery pulsatility index and did not reach statistical significance (aOR 13.34, 95% CI 0.88–201.14), nonetheless, a positive trend was evident. The heterogeneity between the studies may stem from differences in the confounders included in the analyses or variations in the management of PPC cases across the study populations. Given the rarity of fetal and neonatal death in the context of PPC, additional high-quality studies that appropriately adjust for relevant confounders are necessary to draw more reliable and conclusive evidence regarding this association.

A strong positive association emerged between PPC and preterm delivery. Beyond spontaneous etiologies, clinical decisions for iatrogenic preterm delivery may be more frequent in PPC-affected pregnancies due to concerns about deteriorating placental function and fetal status.

Moreover, our data did not demonstrate a significant increase in cesarean delivery rates, while we noted increased risks for low 5 min Apgar score (<7), suspected fetal hypoxia and NICU admission in pregnancies with PPC, highlighting its direct impact on neonatal well-being. Mechanistically, compromised placental perfusion can prompt recurrent fetal hypoxia, leading to heart rate decelerations and reduced neonatal adaptability at birth [30,31]. Notably, while PPC correlated with suspected fetal hypoxia, no significant differences were observed for neonatal resuscitation or GDM. This contrast suggests that not all pregnancies with placental calcification manifest overt metabolic dysfunction or immediate neonatal compromise. However, abnormal fetal heart rate patterns may serve as an early indicator of placental insufficiency, even in the absence of other clinical signs.

Although PPC was strongly associated with SGA and FGR, the pooled estimate for overall birthweight did not achieve statistical significance (mean difference: −187 g). Similarly, while PPC was linked to an increased risk of preterm delivery, the mean gestational age at birth showed only a modest, non-significant reduction (−0.62 weeks). These discrepancies may stem from the limited number of studies and smaller sample sizes available for the analysis of continuous outcomes.

Clinically, PPC shows promise as a candidate variable for integration into next-generation prognostic models that combine ultrasound, Doppler, and biochemical data to better identify high-risk pregnancies. Yet the current evidence base is fragmentary and riddled with residual confounding, precluding immediate clinical implementation. To enhance reproducibility across settings, standardized protocols and dedicated training in the application of the Grannum classification are also needed. Robust, longitudinal cohorts, with systematic adjustment for vascular, metabolic and sociodemographic covariates, together with mechanistic studies that clarify whether calcification is a driver, marker, or innocent bystander of placental senescence are required. Only then can PPC evolve from a descriptive ultrasound finding into a pathophysiology-anchored predictor with real-world clinical utility.

### 5.3. Strengths and Limitations

A key strength of this meta-analysis is the adherence to a robust methodology, including a comprehensive search strategy, strict inclusion criteria focusing on clearly defined PPC and rigorous assessment of risk of bias (via the Newcastle–Ottawa Scale and QUIPS tool). By limiting the analysis to grade 3 placentas before 36^+6^ weeks, we concentrated on a clinically significant presentation of “premature” calcification rather than general placental grading at term.

However, several limitations warrant consideration. First, the included studies varied in design, sample size, and investigated outcomes. Second, heterogeneity was high for certain outcomes, suggesting the influence of unmeasured or residual confounding and potentially affecting the confidence in the pooled estimates. We believe that several factors may contribute to this high heterogeneity, including the different gestational ages at which the measurements were taken, the subjective nature of the Grannum Grading system, and the observational nature of the studies included in the meta-analysis. Third, placental grading remains operator-dependent. Fourth, relatively few studies provided adjusted effect estimates or robust confounder control. Fifth, for several outcomes, such as preterm delivery, neonatal resuscitation, GDM, birthweight and gestational age at birth, only a small number of studies and events were available, thereby limiting the precision of pooled estimates and reducing their reliability. Finally, the sparse data on rare but clinically significant outcomes, such as fetal and neonatal death, restrict the ability to draw conclusions regarding the associations.

## 6. Conclusions

Our findings suggest that PPC may serve as a marker of reduced placental reserve, linked to vascular occlusion, inflammation and suboptimal fetal growth. The strong associations between PPC and adverse outcomes like SGA and preeclampsia highlight the possible value of early detection for enhanced monitoring. Serial fetal growth assessments, Doppler velocimetry and timely delivery decisions could help mitigate risks. Placental grading, despite its inter-observer variability, may complement tools like uterine artery Doppler to improve risk stratification. While not yet a universal component of prenatal assessment, the detection of PPC on ultrasound may serve as an important clinical adjunct to identify pregnancies that warrant intensified fetal surveillance and, when necessary, earlier intervention.

## Figures and Tables

**Table 1:** Characteristics of the included studies.

Study, Year	Study Period	Type	Country	Inclusion Criteria	Exclusion Criteria	Time of Diagnosis	Outcomes	Groups
Chen, 2011 [25]	July 2007 to June 2009	Prospective cohort	Taiwan	Singleton pregnancies	Smoking, alcohol consumption, chronic or pregnancy-induced hypertension (including pre-eclampsia), severe anemia (Hb < 8 g/dL), maternal thalassemia, placenta previa, diabetes (overt or gestational), multiple gestations, or major congenital anomalies detected on antenatal examination	28–32 and 32–36 wks	Birthweight, fetal/neonatal death, gestational age at birth, low 5 min Apgar score, placental abruption, preterm delivery, SGA	Grade 3 vs. no calcification
Chen, 2015 [7]	-	Prospective cohort	Taiwan	Singleton pregnancies	Lost to follow-up, missing data, multifetal gestations, major fetal congenital anomalies, termination before 24 wks, cord accidents, suspected intrauterine infection, or antepartum complications (hypertension, diabetes, placenta previa, severe anemia)	28 wks	Fetal/neonatal death	Grade 3 vs. no calcification
Chitlange, 1990 [5]	-	Prospective cohort	India	Singleton and uncomplicated pregnancies		31–34 wks	FGR, suspected fetal hypoxia, preeclampsia, SGA	Grade 3 vs. 1
Dash, 2021 [4]	October 2017 to September 2019	Prospective cohort	India	All pregnant women attending after 28 wks	Multifetal gestation, congenital anomalies, smoking or alcohol history, previous diabetes or hypertension, or lack of consent	28–36 wks	Birthweight, cesarean section, suspected fetal hypoxia, fetal/neonatal death, FGR, gestational age at birth, low 5 min Apgar score, NICU, preeclampsia, preterm birth	Grade 3 vs. 1, 2
Fouedjio, 2015 [9]	2 January 2012 to 30 July 2013	Prospective cohort	Cameroon	Singleton pregnancies	Sickle cell disease, diabetes, hypertensive disorders before 34 wks, multifetal gestation, or fetal malformations	34–36 wks	Preeclampsia, cesarean section, suspected fetal hypoxia, neonatal resuscitation, SGA, low 5 min Apgar score	Grade 3 vs. 0, 1, 2
McKenna, 2005 [3]	-	Prospective cohort	UK	Singleton pregnancies and known gestational age confirmed by ultrasound at <20 wk.	Multiple pregnancy, maternal medical condition, obstetric complication in a previous pregnancy, obstetric complication before 36 wks in this pregnancy, or known fetal abnormality	36 wks	Suspected fetal hypoxia, neonatal resuscitation, NICU, preeclampsia, SGA	Grade 3 vs. 0, 1, 2
Sersam, 2011 [8]	1 of August 2009 to end of July 2010	Prospective cohort	Iraq	Singleton pregnancies with known gestational age confirmed by ultrasound at <20 wk	Multiple pregnancy, maternal medical condition, obstetric complication in a previous pregnancy, obstetric complication before 36 wks, or known fetal abnormality	36 wks	Suspected fetal hypoxia, meconium, low 5 min Apgar score, preeclampsia	Grade 3 vs. 0, 1, 2
Sun, 2022 [26]	November 2020 to April 2021	Prospective cohort	China	Singleton pregnancy with prenatal care and delivery at the hospital, complete medical history available, and no fetal congenital or chromosomal abnormalities		<37 wks and ≥37 wks	GDM	Grade 3 vs. 0, 1, 2
Siargkas, 2025 [11]	2 January 2018 to 30 December 2023	Retrospective cohort	Greece	Singleton pregnancies carrying a live fetus within the specified gestational age range	Pregnancies with genetic anomalies or major fetal defects, as well as pregnancies lost to follow-up	31–36 wks	FGR, fetal/neonatal death, gestational age at birth, gestational hypertension, preeclampsia, SGA	Grade 3 vs. 0, 1

Abbreviations: FGR, fetal growth restriction; GDM, gestational diabetes mellitus; NICU, neonatal intensive care unit; SGA, small-for-gestational-age; wks, gestational weeks.

**Table 2:** Quality assessment of the included studies according to the Newcastle−Ottawa scale.

First Author, Year	Study Type	S1	S2	S3	S4	C	O1	O2	O3	Total Score
Chen, 2011 [25]	Prospective cohort	a *	a *	a *	a *	a,b **	a *	a *	a *	9
Chen, 2015 [7]	Prospective cohort	a *	a *	a *	a *	a,b **	a *	a *	a *	9
Chitlange, 1990 [5]	Prospective cohort	a *	a *	a *	d	-	a *	a *	a *	6
Dash, 2021 [4]	Prospective cohort	a *	a *	a *	a *	-	a *	a *	a *	7
Fouedjio, 2015 [9]	Prospective cohort	a *	a *	a *	a *	-	a *	a *	a *	7
McKenna, 2005 [3]	Prospective cohort	a *	a *	a *	a *	-	a *	a *	a *	7
Sersam, 2011 [8]	Prospective cohort	a *	a *	a *	a *	-	a *	a *	a *	7
Sun, 2022 [26]	Prospective cohort	a *	a *	a *	a *	-	a *	a *	a *	7
Siargkas, 2025 [11]	Retrospective cohort	a *	a *	a *	a *	a,b **	a *	a *	a *	9

Abbreviations: a, first answer according to Newcastle–Ottawa Scale (NOS); b, second answer according to NOS; d, fourth answer according to NOS; S, selection; C, comparability; O, outcome; *, attribution of a star according to NOS.

**Table 3:** Cumulative results from all the meta-analyses performed.

Risk Factor	Number of Studies	Study Group	Control Group	RR	95% CI	I^2^; *p*-Value
SGA	7	127/597	732/5982	1.99	1.46, 2.70	60%, 0.02
SGA sensitivity analysis	3	88/436	550/3378	1.80	1.13, 2.87	78%, 0.01
FGR	3	66/436	268/3378	2.31	1.30, 4.09	77%, 0.01
Preeclampsia	6	56/342	122/5461	5.27	2.24, 12.40	75%, 0.001
Fetal or neonatal death	3	10/436	19/3378	2.75	0.87, 8.71	40%, 0.19
Preterm delivery	3	56/436	297/3378	2.11	1.00, 4.45	85%, 0.001
Cesarean section	4	177/452	1537/3474	1.26	0.90, 1.78	85%, <0.001
Cesarean section sensitivity analysis	3	175/436	1522/3378	1.30	0.91, 1.85	89%, <0.001
Low 5 min Apgar	4	36/424	65/1539	2.28	1.50, 3.44	0%, 0.43
NICU	4	18/237	83/2752	1.80	1.02, 3.18	0%, 0.75
Neonatal resuscitation	3	73/97	1753/2398	1.04	0.92, 1.16	0%, 0.70
GDM	3	29/188	561/3149	1.17	0.81, 1.70	0%, 0.98
Suspected fetal hypoxia	5	48/301	326/2958	1.71	1.13, 2.56	22%, 0.27
Birthweight	3	587	3164	−187.46	−413.14, +38.21	91%, <0.001
Gestational age at birth	3	373	3378	−0.62	−1.36, +0.11	92%, <0.001

Abbreviations: CI, confidence interval; I^2^ (heterogeneity in meta-analysis); *p*-value, Cochran Q test’s *p*-value; RR, relative risk.

## Data Availability

No original data.

## Supplementary Materials

The following supporting information can be downloaded at: https://www.mdpi.com/article/10.3390/diagnostics15101264/s1, Search strategy.
