# Supplementary material for: The Association of Placental Grading with Perinatal Outcomes: A Systematic Review and Meta-Analysis
Source: Diagnostics (Basel). 2025 May 15;15(10):1264. doi: 10.3390/diagnostics15101264 (PMC12109751; doi:10.3390/diagnostics15101264)
Supplement: Supplementary file 1 [file diagnostics-15-01264-s001.zip › diagnostics-3573146-supplementary.pdf]

Scopus

Search: TITLE-ABS-KEY ("placental maturity" OR "placental calcification" OR "placental aging" OR "placental grading" OR "placental senescence" OR "Grannum")

369 documents found

Pubmed - Medline

Search: (((((Placental Maturity[Title/Abstract]) OR (Placental Calcification[Title/Abstract])) OR (Placental Aging[Title/Abstract])) OR (Placental grading[Title/Abstract])) OR (Placental Senescence[Title/Abstract])) OR (Grannum[Title/Abstract]) Sort by: Most Recent

304 documents found

Cochrane

Search: (((((Placental Maturity[Title/Abstract]) OR (Placental Calcification[Title/Abstract])) OR (Placental Aging[Title/Abstract])) OR (Placental grading[Title/Abstract])) OR (Placental Senescence[Title/Abstract])) OR (Grannum[Title/Abstract])

13 documents found
